# Rethinking the service delivery system of psychological interventions in low and middle income countries

**DOI:** 10.1186/s12888-016-0938-y

**Published:** 2016-07-12

**Authors:** L. K. Murray, M. J. D. Jordans

**Affiliations:** Department of Mental Health, Johns Hopkins Bloomberg School of Public Health, 624 N. Broadway Street, Baltimore, MD 21205 USA; Center for Global Mental Health; Institute of Psychiatry, Psychology and Neuroscience, King’s College London, 16 De Crespigny Park, London SE5 8AF, London, UK; Research and Development Department, War Child Holland, 61G, 1098 Amsterdam, Amsterdam, Netherlands

**Keywords:** Global mental health, Implementation science, Common elements approach, Low resource settings, Health systems

## Abstract

**Background:**

Global mental health is a growing field intricately connected to broader health, violence and economic issues. Despite the high prevalence and cost of mental health disorders, an estimated 75 % of those with need in lower resource settings do not receive intervention. Most studies to date have examined the effectiveness of single-disorder mental health treatments – an approach that may be a significant challenge to scale-up and sustainability in lower resource settings.

**Main body:**

This paper presents a brief overview of the scientific progress in global mental health, and suggests consideration of an internal stepped care delivery approach. An internal stepped care model is one idea of a delivery system, utilizing a common elements approach, where the same provider could navigate between different elements based on severity and type of problems of the client. It is distinct from traditional stepped care models in that clients remain with the same provider, rather than relying on referral systems.

**Conclusion:**

An internal stepped care delivery system based on a simplified common elements approach could be more efficient, scalable, sustainable, and reduce the loss of clients to referrals in lower resource settings.

**Electronic supplementary material:**

The online version of this article (doi:10.1186/s12888-016-0938-y) contains supplementary material, which is available to authorized users.

## Background

Global mental health research and practice is increasing as evidenced by scientific publications, funders, and growing recognition of its importance in the larger scheme of health and productivity [1–3]. Despite the high prevalence and cost of mental health disorders, an estimated 75 % of those with need do not receive intervention [4–7]. Some of the primary barriers to addressing the mental health intervention gap in low and middle-income countries (LMIC) include limited mental health infrastructure or systems, funding, and scarcity of mental health professionals [8–11].

In the last decade, substantial advances have been made in global mental health (for some reviews see [4, 12–14]). A particular growth includes evidence from randomized controlled trials (RCT) which have demonstrated that evidence-based treatments (EBT) targeting common mental disorders, primarily based in cognitive behavioral therapy, can be implemented in LMIC with positive clinical outcomes on mental health symptomatology. Most of these studies have evaluated single-disorder-focused interventions (e.g., Interpersonal Psychotherapy for Depression, IPT; Narrative Exposure Therapy for PTSD, NET) [15–23]. Some of these EBTs are recommended in the recent World Health Organization (WHO) Mental Health GAP (mhGAP) Guidelines [24, 25] as front-line interventions.

Another area of advancement is around the effective implementation of evidence-based interventions in LMIC. For example, due to the shortage of highly trained personnel, most of the studies above used a task-shifting approach or the use of non-professionals with limited, if any, formal mental health training as counselor [26]. Studies have also examined the feasibility and cultural modification of EBT [27–32].

Despite this scientific evidence and policy support from organizations like WHO, very few organizations or countries have been able to scale-up or sustain EBT that have shown to be effective in LMIC. Some literature exists on potential reasons for the lack of scale-up and sustainability of any of these efficacious interventions [11, 33–35]. Frequently mentioned challenges are funding, instability, limited trained personnel, logistics (transport, communications), and lack of time and space for delivery of services [10]. More recently, researchers have suggested that the use of single disorder interventions may be problematic and in the long-term not feasible for scale-up [11, 36, 37].

## Main text

To date, much of the research and implementation of mental health interventions in LMIC has followed an approach similar to some high-income countries (HIC). Mental health in HIC is often trained and delivered via “silos” for certain symptoms and/or severities. For example, a designated clinic may treat a particular problem (e.g., a clinic for substance use), and/or have a group of counselors that each has expertise in treating certain disorders. This “silo” model requires a complex system of triage, referrals, and extensive well-trained personnel. It necessitates accurate assessment, followed by referral to either: (a) specific providers depending on the problem, (b) a provider who had trained on and mastered multiple EBTs, or (c) another clinic that specializes in a particular problem or severity (e.g., anxiety disorder clinic, psychiatric clinic).

Many randomized controlled trials in LMIC that have shown strong effectiveness on mental health symptomatology have evaluated interventions that originated from high-income settings (e.g., IPT; Cognitive Processing Therapy, CPT) and that are disorder-specific (i.e., they were designed and tested to treat one primary disorder) [15–23]. Within these trials, a group of lay providers may be trained to treat depression, for example, but would not know how to address trauma, anxiety or other comorbid or common mental health symptoms. The implication of this is that either: (a) the same lay service providers are required to (eventually) be competent in multiple different interventions to serve at a population level, or (b) each provider would have a specialty to only treat one disorder, and therefore many different providers and referral links would be needed.

We suggest that the segregation of services into “silos”, either related to symptoms/diagnoses or severity, increases the barriers to scale-up and sustainability in LMIC and hinders the ability to reduce the treatment gap. (See Additional file 1: Figure S1) First, having disorder specific interventions suggests (and requires to a degree) a “fit” into Western diagnostic categories as exemplified in the Diagnostic and Statistic Manual (DSM) and International Classification of Disorders (ICD), which some argue is questionable cross-culturally [31, 38, 39]. Second, comorbidity is the rule – not the exception – along with other problems that can affect the course of intervention (e.g., relationship problems), although these may not meet a diagnostic category. As Weisz (2015) [39] puts it, “stated simply, most EBTs are more narrowly focused, and more linear in design, than the everyday clinical practice they are designed to enhance”. Third, with task-shifting being advocated as a strategy to address limited human resources in LMIC [34, 40], it is questionable whether this approach is feasible for ultimate scale-up and sustainability since it would require either large numbers of lay providers each focused on a particular mental health problem or that individuals with limited education learn multiple EBT. Both of these would be difficult with task-sharing. Finally, silo’ed care requires options for referral to other providers or settings that are rarely available in many lower resource settings.

### A different delivery system conceptualization

To more effectively reap the benefits of science to practice and scale up of EBTs, a different mental health systems approach may be needed in certain contexts. We suggest consideration of an “*internal* stepped care model” that allows for the same non-professional service provider (or number of providers) to navigate between different intervention elements based on the severity (i.e. continuous from low to moderate-to-severe) and type of problems (i.e. diverse symptom clusters focused on common mental disorders) of the client.

#### Based on navigating common elements

Common elements approaches, also known as transdiagnostic, are increasingly being used, studied and suggested as an alternative way to approach mental health scale up [37, 41–44]. A common elements approach is derived from research showing that most EBTs are actually comprised of many of the same elements or components [42]. In this way, *elements* are taught (rather than a particular manual), including how to combine them to use for different symptoms and severity levels [43, 44]. Therefore, providers need be trained in only *one* approach (consisting of common elements and their flexible use), and each provider would be able to treat a range of presenting problems as well as varying severities of common mental health problems depending on the transdiagnostic approach taught (e.g., depression, trauma, anxiety, externalizing symptoms, substance use).

Data on effectiveness of common element approaches is emerging both in high-income countries and LMIC. In the United States and Europe, studies are showing positive results across both adult [45–49] and child populations [50]. These approaches are performing at or better than single disorder treatments. However, this work in HIC has been done by mental health professionals. Thus, a significant question about the use of common elements approaches in LMIC is if non-professional providers can be trained to select elements based on the needs of a client, both in terms of severity and type of problem and deliver them adequately. The desire to use a common elements approach for scale-up and sustainability would be a mute point if non-professional providers with limited education could not learn the multiple elements included in the approach, and know how to put them together for a range of client presentations.

A modular common elements approach was developed specifically for LMIC that was based off current research in the United States with MATCH and the Unified Protocol (UP), [45, 50] but with a reduced number of elements and simplified decision rules to account for the training of non-professionals (Common Elements Intervention Approach or CETA) [37]. Briefly, CETA developers utilized distillation research [51] and consultation with developers of multiple evidence-based treatments in an attempt to choose the most frequently used elements, and those that seemed to be the “mechanism of action”. Two trials (Iraq and Thailand/Myanmar border) on adult populations that were trauma-affected were completed with CETA - both showing strong effectiveness on symptoms of depression, trauma and anxiety with effect sizes >1) [52, 53]. In Iraq, CETA performed better than single disorder treatments. One open trial of CETA for youth was completed in Ethiopia with significant results [54]. Although more studies are needed, these studies suggest that: (1) para-professionals are able to learn a simplified common elements approach (inclusive of 9 elements only) with an apprenticeship model of training and ongoing supervision [55], and, (2) that the elements chosen for CETA were collectively as effective or more for comorbid presentations in comparison to single disorder treatments or control conditions.

It is important to note that CETA, MATCH, CBT-E, and UP are examples of common elements approaches that could make an internal stepped care model possible. Although these transdiagnostic treatments utilize CBT-based elements, other elements could be utilized, as long as there was evidence supporting them from rigorous research in accordance with current guidelines on best practices [4, 25].

**Fig. 1:**
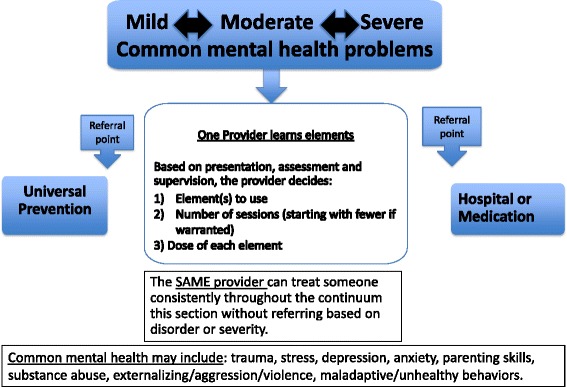
Internal Stepped Care Approach

A common elements approach allows for an internal stepped care model (Fig. 1), which is an attempt to address some of the challenges of implementation, reach, scale-up and sustainability of mental health programs in LMIC. In practice, a service provider could be trained in a common elements approach and learn how to put elements together for different common mental health disorders. Upon assessment, this one provider could decide to start with a smaller set of elements due to lower symptoms, and only add elements if there were sustained problems. Alternatively, upon assessment, a provider may note moderate to severe symptoms and choose an order of elements indicated for the presenting problems based on existing EBT. In either case the service provider would be able to add elements and/or dose of elements based on need and client response. This flexibility, within fidelity to the evidence base, allows the same single provider to address a wide range of problems and severities, and provide only what the client needs based on symptom presentation throughout.

To our knowledge, CETA is the only common elements approach that has been tested in LMIC. Nonprofessionals were successfully trained in the choice patterns discussed above by learning to gleam information from three “data points” throughout treatment: 1) assessment form (client self-report), 2) what the client does and says directly, and 3) consultation with a supervisor. This helps determine what the main problems are of the client. There was not a focus on “diagnoses” as one would in Western psychiatry. Changes could be made to the element choice and dose based on these three information sources throughout treatment. This is one example of how non-professionals could be taught. More research is needed on how well and with how much support non-professional counselors are able to adequately assess the severity and core problems to address in a range of clients.

### How is this different from a stepped care approach?

Stepped care models advocate moving from lower-intensity and least restrictive interventions to higher-intensity and more restricted access interventions based on the lack of desired effect of the previous level of care, [56] generally moving from one service provider or organization to the next. One challenge with this type of stepped care approach in lower-resourced settings is the inherent assumption that there *is* a “next step” if someone does not respond to the first step of intervention. In most LMIC, there are not enough mental health professionals or even lay providers trained in any EBT that could offer services for moderate to severe common mental disorders. In our proposed model, this transfer still happens but *within one provider* utilizing one approach. The individual could still begin by providing a brief intervention that requires fewer health care resources, but would then be capable of providing ongoing services if the desired intervention benefits were not obtained. The internal stepped care model reduces the need for different groups or levels of provider types, and different specialized settings, which may not be possible in some LMIC settings.

A related challenge with a traditional stepped care model is that it usually includes referral points (from low to high, or across problem area). Every referral point where a client needs to change providers or locations increases the likelihood of them being “lost”. Imagine a depressed client who rarely leaves the house, finally making it into a clinic in a low resource area. After a likely long wait and being screened, the lay provider says they do not treat these types of symptoms (e.g., a primary health care worker is insufficiently trained to provide psychological treatments). Although a referral is made, it is quite likely that this client will not make the next referral appointment perhaps due to depressive symptoms, or other reasons such as distance or stigma.

### Next steps to consider

There are numerous research questions that could help determine whether an internal stepped care delivery system is truly beneficial and feasible. First, understanding more about what common elements are needed and used, at what levels of symptomatology, with which symptom clusters, and the doses needed for symptom reduction would further refine the use of common elements approaches. Secondly, although research suggests that clinical decision-making within a common elements approach is possible for para-professionals, [52, 53] this model adds variation in symptom presentation and severity beyond these particular studies. Evaluation of the training and supervision needed for an internal stepped care delivery system will be critical. This might include evaluation of key indicators of competency [57] or capacity of trainees in clinical decision making, as well as the amount of resources needed to obtain “adequate” skill levels. Third, common elements approaches have yet to be evaluated in groups – which is a delivery system of interest in many LMIC. Learning how flexibility of element choice and dose fits into group models will need to be studied. Finally, this internal stepped care model is a service delivery framework that addresses some of the known challenges with broader implementation and sustainability of effective mental health interventions. However, it will be important to assess what settings this may or may not work within. Implementation constructs including cost-effectiveness, feasibility, acceptability, and appropriateness will need to be assessed, as well as who provides services and to what degree, to what types and severity of populations.

## Conclusions

The field of global mental health is at an important crossroad where there is now increasing evidence to suggest that some EBTs are effective and feasible in low resource settings – and yet there is limited scale-up or sustainability of these. This means although we have growing information on what works to alleviate mental health suffering, most populations in need are not receiving these services. Thinking through implementation methods is critical. It is clear that the costs of implementing, with fidelity and thus effectiveness, even *one* EBT are enormous. Consequently it is important to be selective when choosing an EBT for implementation that fits within the context and meets the needs of the population. We have suggested that mimicking mental health care delivery approaches that are commonplace in higher resource settings may not be the most sustainable in some LMIC where funding, personnel and infrastructure are lacking. An internal stepped care model is one idea of a delivery system, utilizing a common elements approach, where the same provider could navigate between different elements based on severity and type of problems of the client. This delivery system could be more efficient, scalable, sustainable, and reduce the loss of clients to referrals (existent or non-existent) in LMIC. This certainly does not solve all the challenges found in scaling up global mental health and further research is required to evaluate this strategy in the future.

## Abbreviations

CBT, cognitive behavioral therapy; CETA, common elements treatment approach; DSM, Diagnostic and Statistic Manual; EBT, evidence-based treatment; ICD, International Classification of Disorders; IPT, Interpersonal Psychotherapy for Depression; LMIC, low and middle-income country; RCT, randomized control trial

## Additional file

Additional file 1: Figure S1. Example system flow with “silo’ed” treatments. (PPTX 113 kb)
