# Supplementary material for: Rethinking the service delivery system of psychological interventions in low and middle income countries
Source: BMC Psychiatry. 2016 Jul 12;16:234. doi: 10.1186/s12888-016-0938-y (PMC4941014; doi:10.1186/s12888-016-0938-y)
Supplement: Additional file 1: Figure S1. — Example system flow with “silo’ed” treatments. (PPTX 113 kb) [file 12888_2016_938_MOESM1_ESM.pptx]

## Slide 1
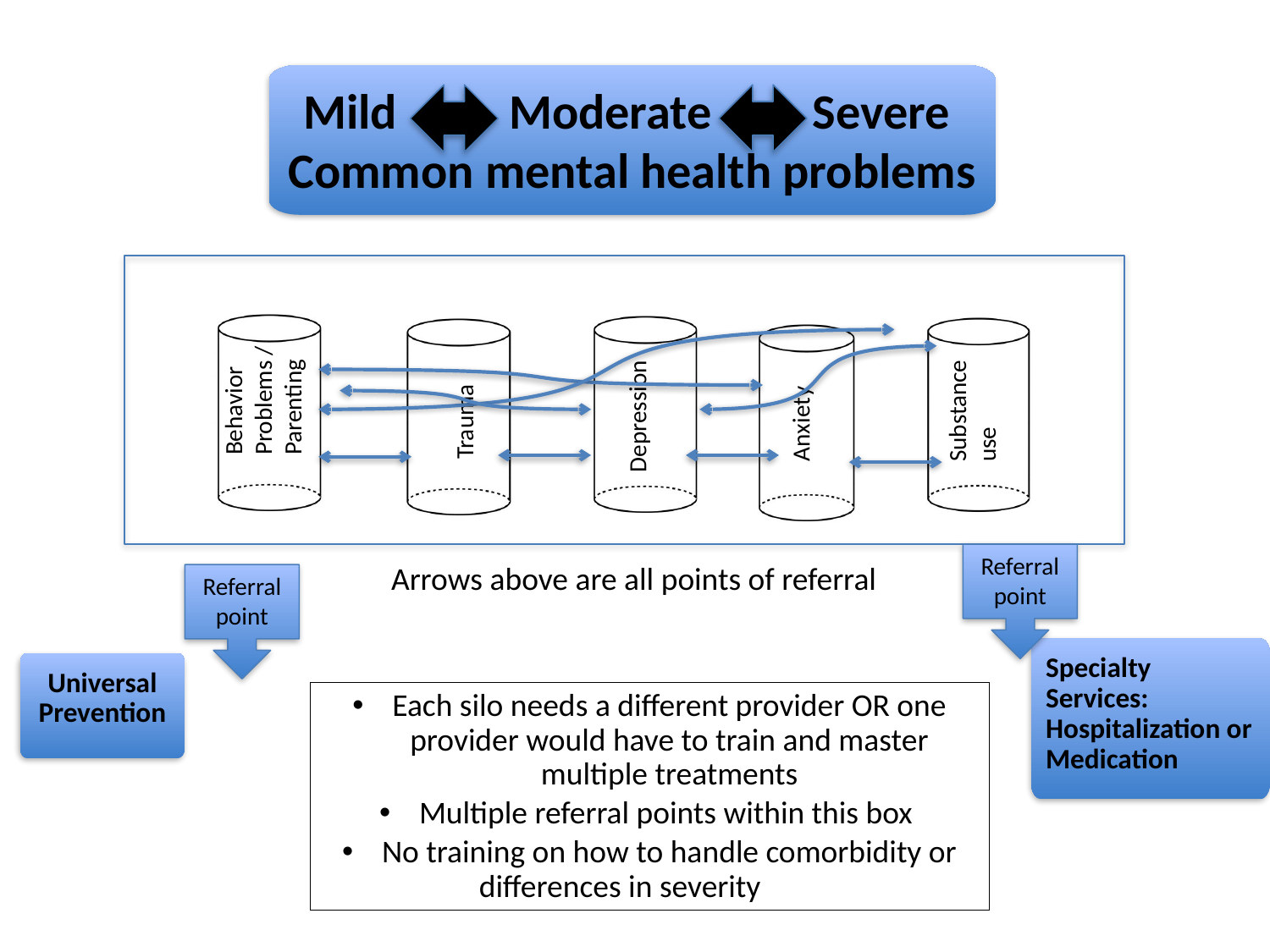

Mild Moderate Severe
Common mental health problems
Behavior Problems / Parenting
Depression
Substance use
Trauma
Anxiety
Referral point
Arrows above are all points of referral
Referral point
Specialty Services: Hospitalization or Medication
Universal Prevention
Each silo needs a different provider OR one provider would have to train and master multiple treatments
Multiple referral points within this box
No training on how to handle comorbidity or differences in severity
